# Binary symbolic dynamics analysis to detect stress-associated changes of nonstationary heart rate variability

**DOI:** 10.1038/s41598-020-72034-2

**Published:** 2020-09-22

**Authors:** Conrad Spellenberg, Peter Heusser, Arndt Büssing, Andreas Savelsbergh, Dirk Cysarz

**Affiliations:** 1 Medical Anthropology, Institute of Integrative Medicine, Witten/Herdecke University, Gerhard-Kienle-Weg 4, 58313 Herdecke, Germany; 2 Quality of Life, Spirituality and Coping, Institute of Integrative Medicine, Witten/Herdecke University, Herdecke, Germany; 3 Division of Functional Genomics, Center for Biomedical Research and Education ZBAF, Witten/Herdecke University, Witten, Germany; 4 Integrated Curriculum for Anthroposophic Medicine, Witten/Herdecke University, Herdecke, Germany; 5 Physiologic Rhythm Research, Witten/Herdecke University, Herdecke, Germany

**Keywords:** Physiology, Biomedical engineering, Autonomic nervous system, Human behaviour

## Abstract

Psychological stress may have harmful physiological effects and result in deteriorating health. Acute psychological stress acts also on cardiac autonomic regulation and may lead to nonstationarities in the interbeat interval series. We address the requirement of stationary RR interval series to calculate frequency domain parameters of heart rate variability (HRV) and use binary symbolic dynamics derived from RR interval differences to overcome this obstacle. 24 healthy subjects (12 female, 20–35 years) completed the following procedure: waiting period, Trier Social Stress Test to induce acute psychological stress, recovery period. An electrocardiogram was recorded throughout the procedure and HRV parameters were calculated for nine 5-min periods. Nonstationarities in RR interval series were present in all periods. During acute stress the average RR interval and SDNN decreased compared to rest before and after the stress test. Neither low frequency oscillations (LF), high frequency oscillations (HF) nor LF/HF could unambiguously reflect changes during acute stress in comparison to rest. Pattern categories derived from binary symbolic dynamics clearly identified acute stress and accompanying alterations of cardiac autonomic regulation. Methods based on RR interval differences like binary symbolic dynamics should be preferred to overcome issues related to nonstationarities.

## Introduction

Psychological stress can be defined as a psychological state that occurs when “an individual perceives that environmental demands tax or exceed its adaptive capacity”^1,2^. If the stressor’s influence exceeds the individual coping capacity due to intensity or duration, psychological stress can have harmful physiological effects. Therefore, stress is reasonably suspected to function as an important co-factor for the genesis and maintenance of a large number of acute and chronic diseases of almost each physiological system of the organism, such as cardiovascular, respiratory, gastroenterological, autoimmune and inflammatory, metabolic, neurological, mental and psychiatric diseases^3–7^. Functioning as a co-factor, this also includes diseases of all severities, ranging from the common cold^3^ up to severe diseases, such as atherosclerosis^4^, coronary heart disease, rheumatoid arthritis, peptic ulcer diseases, ulcerative colitis and even malignant diseases such as breast cancer^7^, as well as asthma^6^, diabetes mellitus^5^, or several more. In particular, such harmful stress effects have already been investigated for the field of neuronal diseases as well as mental and psychiatric disorders such as schizophrenia or major depressive disorders (MDD)^7^.

To cope with acute psychological stress, the organism initiates several physiological, biochemical and molecular processes that can be conflated as the psychological stress response^8^. Furthermore, epigenetic regulation and gene expression are also altered^9^. Here, we focus on cardiac autonomic regulation of the acute stress response elicited by the Trier Social Stress Test (TSST), a standardized experimental social stress test^10–12^. In terms of physiological aspects, the acute stress response initiates two different processes; the sympathetic activation of the autonomic nervous system (ANS) as the ‘alarm’ response providing short-term effects and the activation of the hypothalamic–pituitary–adrenal (HPA) axis as a delayed response providing long-term effects^13,14^. The sympathetic activation causes an increased release of transmitters and hormones in the central and peripheral nervous system^4,13^. This leads to changes in the organism that are necessary to facilitate a ‘fight, fright or flight’ response, such as elevating the metabolic rate, the blood pressure and respiration and increasing the blood flow to the heart and skeletal muscle^13^. Activation of the HPA-axis as the second stage, provides energy for a longer period of time affecting the individual’s behavioral, neuronal and hormonal response to stress^13^.

Especially the ‘alarm’ responses of acute stress, for example, the sympathetic activation and parasympathetic withdrawal of autonomic nervous functioning during acute stress, are suggested to be assessable by parameters of heart rate variability (HRV)^15,16^. Sympathetic activity of cardiac autonomic regulation is often calculated using low frequency (LF) power of spectral analysis of HRV whereas parasympathetic activity may be assessed using high frequency (HF) power or the root of the mean squares of differences between adjacent RR intervals (RMSSD) in the time domain^17^.

Methodological issues are rarely addressed in the context of the assessment of stress-related changes by means of frequency domain parameters of HRV. The application of power spectral analysis to a physiological time series requires stationary conditions of the time series. I.e., the underlying physiological system producing the RR interval series should be as constant as possible to meet this condition. Especially during stress related responses of autonomic regulation this prerequisite is rarely met. As a solution, the analysis of short term recordings using durations considerably shorter than the standard of 300 s was recently suggested^15^. However, the shorter the time series the more the different HRV measures deviate from the calculations carried out over the 300 s duration^18,19^. E.g. the amount of LF oscillations is likely to be underestimated and spectral leakage gets more prominent for shorter timer series^20^. Hence, especially the cardiac sympathetic response to stress cannot be reliably assessed using the LF component. The bias in the calculation of LF may then lead to less pronounced differences in the time course of the acute stress response.

The quantification of HRV by means of parameters derived from symbolic dynamics analysis provides solutions to overcome two of the main obstacles: (1) the coarse grained description of the RR interval series may be chosen in such a way that the nonstationarity condition does not apply anymore. (2) Appropriately chosen parameters reflecting the variability within the symbolic series require fewer data to yield proper results. It has been shown that a binary description based on the differences between adjacent RR intervals still contains sufficient information to capture the alterations of cardiac autonomic activity during a graded head-up tilt test procedure^21,22^. The occurrence of specific pattern categories could be linked to parasympathetic and sympathetic functioning of cardiac autonomic regulation.

In this study, we first explore the stationarity of RR interval series as a prerequisite for the calculation of frequency domain parameters. Data captured from 24 healthy participants during acute stress induced by the Trier Social Stress Test (TSST) is used for this purpose. Perceived stress is quantified using a visual analogue scale (VAS). The RR interval series is used to determine the existence of nonstationarities. HRV is quantified using time and frequency domain parameters as a standard set of HRV parameters. Furthermore, three pattern categories derived from binary symbolic dynamics are used to quantify HRV. The time course of each parameter is used to gain insight in alterations of cardiac autonomic regulation during acute stress.

## Results

**Figure 1:**
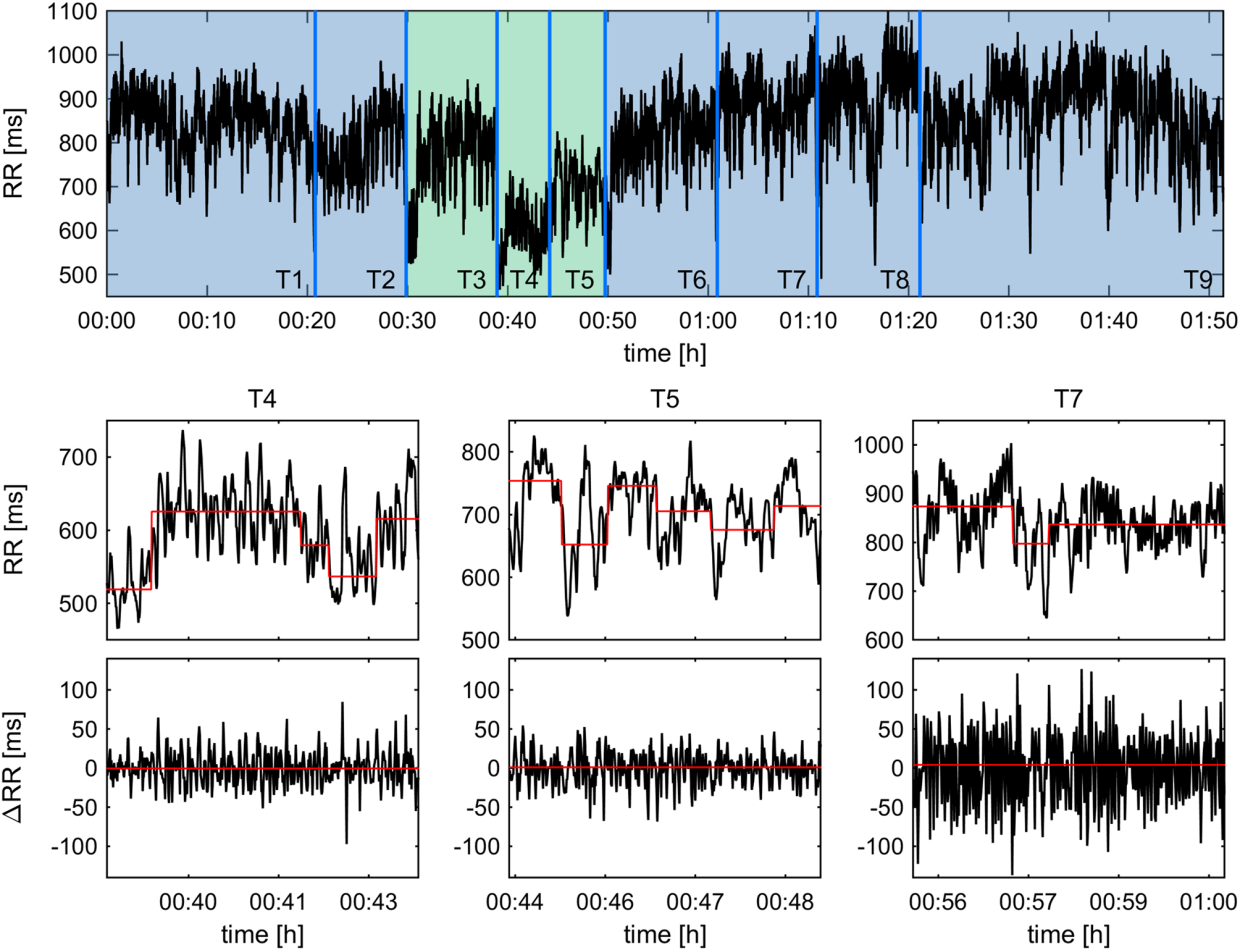
Example of nonstationarities in the RR interval series. Top diagram: Example of the RR interval series during the entire procedure. The blue vertical lines indicate the beginning/end of each period as denoted in Fig. 2. Short RR intervals during periods T4 and T5 indicate stress. Middle row: 5-min RR interval series during the stress periods T4, T5 and recovery period T7. The red lines indicate the median RR interval during stationary segments found by the heuristic segmentation algorithm. Bottom row: differences of successive RR intervals of the analysis periods T4, T5 and T7. The straight red lines indicate that each series is stationary.

The stress test procedure comprised a waiting period (30 min), the actual stress test period (20 min), and a recovery period (60 min), cf. Fig. 1. Nine 5-min periods were analyzed: T1, T2: waiting period; T3–T5: TSST (T3: speech preparation, T4: speech delivery, T5: mathematical task); T6–T9: recovery period.

### Visual analogue scale (VAS)

Across the procedure, changes in the VAS-scores as a marker for the subjectively perceived stress, were significant (p_Skillings-Mack_ < 0.001). The median VAS-score increased from the waiting period T1 (5) to the TSST test-period, where the maximum was observed at the end of the TSST at T5 (59) directly after the arithmetic task. Subsequently, the VAS-scores decreased continuously from T5 to T9 (end of recovery period) back to the level baseline (Table 1). Correspondingly, the VAS-score at T4 and T5 were statistically different from all other analysis periods before and after the stress exposure. Gender differences were not observed.

**Table 1:** Visual analog scale and heart rate variability parameters in the course of the experimental procedure.

	T1	T2	T3	T4	T5	T6	T7	T8	T9
VAS^#^	5	6	39	36	59	16	10	6	3
	1–10	2–12	20–52	22–57	30–78	10–31	2–17	0–10	0–8
	-	T1	T1, T2	T1, T2, T3	T1, T2, T4	T1, T2, T3, T4, T5	T1, T2, T3, T4, T5, T6	T2, T3, T4, T5, T6, T7	T2, T3, T4, T5, T6, T7
RR intervall (ms)***	855	813	750	616	672	839	882	853	885
	730–920	724–887	699–793	573–682	641–771	785–925	815–935	819–956	789–960
	-	-	T1, T2	T1, T2	T1, T2, T4	T1, T2, T3, T4, T5	T1, T2, T3, T4, T5, T6	T1, T2, T3, T4, T5, T6	T1, T2, T3, T4, T5, T6
SDNN (ms)***	66	76	72	61	58	80	72	81	73
	52–95	68–89	51–94	48–75	45–72	64–104	62–97	62–109	59–100
	-	-	-	T1, T2, T3	T1, T2, T3	T4, T5	T4, T5	T4, T5	T1, T4, T5
RMSSD (ms)***	37	36	33	21	25	48	44	46	40
	30–49	29–49	27–49	16–28	16–35	34–62	35–55	32–58	30–56
	-	-	-	T1, T2, T3	T1, T2, T3	T1, T2, T3, T4, T5	T1, T2, T3, T4, T5	T1, T2, T3, T4, T5	T1, T2, T3, T4, T5, T6
LF(ln ms^2^)*	7.11	7.45	7.36	7.54	6.93	7.60	7.41	7.22	7.34
	6.65–7.77	7.17–7.74	7.13–7.91	6.90–7.86	6.47–7.82	6.98–7.99	6.99–8.05	6.96–7.80	6.68–8.36
	-	-	-	-	T2, T3, T4	T1, T5	-	T5	T5
HF (ln ms^2^)***	5.94	6.12	6.36	6.06	5.51	6.70	6.77	6.76	6.44
	5.55–6.67	5.63–6.57	5.86–7.04	5.44–6.52	5.17–6.37	6.01–6.99	6.04–6.97	5.76–7.04	5.77–6.96
	-	-	T2, T3	T3	T1, T2, T3, T4	T1, T2, T4, T5	T1, T2, T4, T5	T1, T2, T4, T5	T1, T2, T4, T5
LF/HF**	1.14	1.41	0.82	1.36	1.23	1.05	0.89	0.63	1.04
	0.47–1.69	0.91–1.79	0.57–1.58	1.06–1.94	0.79–1.95	0.56–1.21	0.40–1.38	0.28–1.58	0.68–1.41
		T1	T2	-	-	T2, T5	T2, T4, T5	T2, T4, T5	T2
LF%***	27.6	34.3	32.2	51.4	33.8	35.2	29.3	28.3	34.0
	22.8–41.5	24.0–41.6	22.7–42–0	39.7–57.1	29.2–46.7	23.0–42.9	22.8–36.8	18.6–36.5	23.7–38.4
	-	-	-	T1, T2, T3	T4	T4	T4	T4	T4
HF%*	9.7	8.8	12.0	12.3	7.4	10.9	12.6	11.0	11.6
	6.3–14.4	6.0–12.3	5.9–18.5	8.5–15.6	5.8–14.1	7.8–16.7	8.1–20.9	7.1–17.7	6.5–17.6
	-	-	T2	T2	-	T2	T2		T2

The first row shows the median, the second row shows the 25%- and 75%-percentile. The lowermost row of each parameter lists significant differences to other times (*p* < 0.05).

^#^*p*_Skillings-Mack_ < 0.001, *p_Friedman_ = 0.05, **p_Friedman_ < 0.01, ***p_Friedman_ < 0.001.

### Nonstationarities in the RR interval series

Figure 1 shows an example of a RR interval series of one subject throughout the procedure. The stress response on the RR interval series during analysis periods T4 and T5 reflects subjectively perceived stress as quantified by VAS. The decrease of RR intervals indicates elevated physiological stress. Qualitatively, in this particular example the recovery from the stress exposure takes a few minutes as can be seen in the transition from T5 to T6 which shows a lengthening of the RR intervals.

Stationary segments of the RR interval series *RR*_*i*_ during stress periods T4, T5 and recovery period T7 are depicted in the middle row of Fig. 1. As expected, the stress response during T4 and T5 leads to several short stationary segments indicating that each entire RR intervals series during T4 and T5 is nonstationary. Unexpectedly, also the recovery period T7 shows three stationary segments, i.e. the RR interval series is nonstationary also during T7. These findings are supported by the results of the Restricted weak stationarity (RWS) test because the randomly chosen subsequences show different means (*p* < 0.001) and different variances (*p* < 0.05) during T7. The entire group also showed a varying amount of stationary segments in the course of the procedure (p_Friedman_ < 0.01). A median of four stationary segments was found in T1, T2, T5, T7, T8 and T9. T3 and T4 had five stationary segments whereas T6 had only three segments. Accordingly, the RWS analysis also indicated nonstationarities in the different analysis periods: in 205 out of 216 analysis periods the average RR interval varied significantly in the randomly chosen subsequences indicating nonstationarities. The variance varied significantly in the subsequences in 200 out of 216 analysis periods. Gender differences were not observed. In contrast, the series of differences of successive RR intervals Δ*RR*_*i*_ was stationary in all cases. I.e. none of the series of differences was segmented neither did the RWS analysis indicate nonstationarities. Consequently, the analysis of binary patterns was not influenced by nonstationarities.

### Heart rate variability

The increase in perceived stress during the stress test was accompanied by physiological stress as assessed by parameters of HRV. Stress decreased the median RR interval compared to the waiting period before and the recovery period after the stress test (T1: 855 ms, T4: 616 ms, T9: 885 ms, p_Friedman_ < 0.001; see Table 1; Fig. 1). During stress period T5 the median RR interval increased compared to T4 (T5: 672 ms). The decrease of the median RR interval during the stress test was accompanied by a decrease of SDNN indicating a lower HRV during stress compared to the waiting period and the recovery period (T1: 66 ms, T4: 61 ms, T5: 58 ms, T9: 73 ms, p_Friedman_ < 0.001). And also the RMSSD decreased as the median RR interval decreased (T1: 37 ms, T4: 21 ms, T5: 25 ms, T9: 40 ms, p_Friedman_ < 0.001). RMSSD was higher during the recovery period compared to the waiting period (*p* < 0.05). Furthermore, RMSSD was higher at the beginning of the recovery period compared to the end of this period (T6: 48 ms, T9: 40 ms, *p* < 0.05).

With respect to gender differences only the median RR interval during stress period T4 showed a difference: male subjects had a higher median RR interval compared to female subjects (645 ms vs. 582 ms, *p* < 0.05). However, this difference did not lead to gender differences in any HRV parameter in the time and frequency domain nor did it lead to gender differences in the symbolic dynamics parameters.

The frequency domain parameters reflected the perceived stress to a lesser extent. LF was lowest during the stress test compared to the second part of the waiting period and compared to the end of the recovery period (T2: 7.45 ln ms^2^, T5: 6.93 ln ms^2^, 7.34 ln ms^2^, p_Friedman_ < 0.05). However, the first part of the waiting period (T1) and the middle of the recovery period (T7) were not different from the stress test (T5). HF was also lowest during stress test compared to the waiting period and the recovery period (T1: 5.94 ln ms^2^, T5: 5.51 ln ms^2^, T9: 6.44 ln ms^2^, p_Friedman_ < 0.001). HF was higher during the recovery period compared to the waiting period (*p* < 0.05). The ratio LF/HF did not show an unequivocal increase during the stress test nor was it systematically lower during the waiting period or the recovery period. LF/HF was lowest during the recovery period and highest during the waiting period (T8: 0.63, T2: 1.41, p_Friedman_ < 0.01). LF% was highest during the stress period compared to all other periods (T4: 51.4, T1: 27.6, T6: 35.2, p_Friedman_ < 0.001). HF% did not show an unequivocal decrease during the stress test. Instead, it was lowest during waiting period T2 compared to waiting period, stress test and recovery period (T2: 9.7, T3: 12.0, T4: 12.3, T6: 10.9, T7: 12.6, T9: 11.6; p_Friedman_ < 0.001).

The symbolic dynamics parameters were also able to reflect changes in the course of the experimental procedure. The pattern category P0V% derived from acceleration and deceleration of RR intervals was significantly lower during the waiting period and recovery period compared to the stress test (T1: 24.4, T4: 44.4, T9: 24.5, p_Friedman_ < 0.001, see Table 2). At the same time pattern categories P1V% and P2V% were higher during the waiting period and recovery period compared to the stress test (P1V%: T1: 60.0, T4: 44.3, T9: 58.1, p_Friedman_ < 0.001; P2V% T1: 15.7, T4: 10.7, T9: 15.9, p_Friedman_ < 0.001). The symbolic parameters using a threshold also reflected the course of the experimental procedure. Pattern category P0V_τ_% was low during the waiting period and the recovery period and increased during the stress test (T1: 38.0, T4: 76.3, T8: 34.3, p_Friedman_ < 0.001). Pattern categories P1V_τ_% and P2V_τ_% were lowest during the stress test compared to the waiting period and the recovery period (P1V_τ_%: T3: 38.8, T4: 17.4, T6: 43.6, p_Friedman_ < 0.001; P2V_τ_%: T1: 19.7, T4: 5.2, T8: 21.3, p_Friedman_ < 0.001).

**Table 2:** Symbolic dynamics parameters in the course of the experimental procedure.

	T1	T2	T3	T4	T5	T6	T7	T8	T9
P0V%***	24.4	29.1	32.2	44.4	37.0	28.2	24.8	23.1	24.5
	19.6–32.4	26.3–35.8	27.0–38.3	37.4–50.0	31.7–46.8	22.3–33.2	14.3–28.3	16.2–28.8	21.1–30.9
	-	T1	T1	T1, T2, T3	T1, T2	T3, T4, T5	T2, T3, T4, T5, T6	T2, T3, T4, T5	T2, T3, T4, T5, T7
P1V%***	60.0	54.3	55.8	44.3	52.1	56.8	58.6	62.9	58.1
	54.5–65.9	51.3–60.2	49.5–60.6	39.2–49.7	42.9–54.9	52.4–60.6	55.4–67.2	53.4–67.0	53.4–62.4
	-	T1	T1	T1, T2, T3	T1, T2, T3	T4, T5	T2, T3, T4, T5	T2, T3, T4, T5	T2, T3, T4, T5
P2V%***	15.7	15.4	12.9	10.7	10.9	14.9	14.9	15.0	15.9
	12.5–18.3	12.4–18.2	9.1–14.5	8.8–13.3	9.3–14.2	11.9–18.2	12.5–18.7	11.2–16.6	13.1–20.3
	-	-	T1, T2	T1, T2	T1, T2	T3, T4	T3, T4, T5, T6	T3, T4, T7	T3, T4, T5
P0V_τ_%***	38.0	46.6	45.1	76.3	71.1	35.5	37.0	34.3	38.4
	31.0–50.4	28.4–58.8	36.0–57.3	62.2–89.1	45.2–91.2	28.7–52.8	26.4–45.6	29.2–46.8	31.5–49.0
	-	-	-	T1, T2, T3	T1, T2, T3	T1, T2, T3, T4, T5	T3, T4, T5	T3, T4, T5	T3, T4, T5
P1V_τ_%***	38.6	38.4	38.8	17.4	21.9	43.7	43.2	43.3	40.3
	33.3–45.4	31.3–48.0	30.8–44.5	8.4–28.5	5.9–36.6	33.5–49.2	37.6–47.3	34.4–47.7	34.5–46.8
	-	-	-	T1, T2, T3	T1, T2, T3	T1, T3, T4, T5	T1, T3, T4, T5	T1, T3, T4, T5	T3, T4, T5, T6
P2V_τ_%***	19.7	14.8	15.9	5.2	6.2	20.0	19.6	21.3	19.0
	15.9–25.0	12.9–21.2	11.5–21.7	2.7–9.5	3.0–17.5	14.6–22.0	15.0–25.9	15.3–24.7	15.8–23.9
	-	-	-	T1, T2, T3	T1, T2, T3	T4, T5	T2, T3, T4, T5	T4, T5	T4, T5

The first row shows the median, the second row shows the 25%- and 75%-percentile. The lowermost row of each parameter lists significant differences to other times (*p* < 0.05).

***p_Friedman_ < 0.001.

## Discussion

The impact of different kinds of stress (e.g. mental stress, psychosocial stress) on physiological functioning such as the heart rate and heart rate variability has been investigated in numerous studies^23–29^. The Trier Social Stress Test as a standardized psychosocial stress procedure has also been investigated with respect to changes of HRV during the procedure^15,16,30^. However, issues arising from nonstationarities in the analyzed time series caused by the stress testing procedure are rarely addressed^31^. In this study, we showed that the stress testing procedure imposed nonstationarities on the RR interval series. We observed nonstationarities in the RR interval series during the stress test. The nonstationarities during stress are obviously caused by the changing demands (‘nonstationary’ conditions) during the stress procedure giving rise to changes of cardiac autonomic regulation and, hence, irregular trends in the time series. Surprisingly, nonstationarities were also observed during quiet rest in the waiting and recovery period although this condition would be called a ‘stationary’ condition. These nonstationarities in the time series may also have been caused by irregular trends arising from e.g. different depths of relaxation. I.e. although the resting condition seems to be ‘stationary’ it may still change during its course. We note that we did not control for breathing nor did we give any instructions with respect to relaxation during the resting periods. However, acute mental stress may lead to alterations of breathing patterns^23^ and also cardiorespiratory interaction^32^. Hence, alterations of breathing patterns during acute mental stress and speech may have contributed to alterations of cardiac autonomic regulation and may also lead to nonstationarities. Furthermore, very low frequency fluctuations linked to e.g. vagal baroreflex sensitivity may also lead to nonstationarities of the RR interval series^33^ because the shortest segments contained 40 RR intervals approximately equivalent to the threshold between very low frequency and low frequency oscillations in the frequency domain.

The Fourier transformation requires stationarity of the underlying time series. Hence, nonstationarities in the RR interval series have an impact on the calculation of the HRV parameters in the frequency domain. A comparison between parameters calculated from Fourier analysis and parameters calculated from the wavelet transformation, which can be used for nonstationary time series, showed only small differences^34^. Nevertheless, the differences may attenuate the variance across the stress testing procedure for parameters of the Fourier transformation. The LF parameter showed relatively little variance across the waiting, stress and recovery periods. During stress period T5 LF was lowest. However, the decrease of LF is contrary to what would be expected: the stress periods T4 and T5 elicit sympathetic activation as reflected by the decrease of the median RR interval and SDNN. At the same time parasympathetic activity decreased as indicated by RMSSD^17^. As LF is affected by sympathetic as well es parasympathetic influence, LF in this particular case does not reflect the sympathetic activation but seems to reflect only the decrease of parasympathetic activity. LF% as an equivalent to LF expressed in normalized units should reflect solely sympathetic activity^17^. Indeed, LF % showed an increase during T5 indicating the sympathetic activation correctly. Of note is that during a tilt testing procedure eliciting cardiac sympathetic activation the LF parameter also performed worse compared to LF%^35^. Hence, LF% may be better suited as a parameter reflecting sympathetic activation although it did not reflect the increase in stress during stress period T4.

HF was consistently lower during T5 compared to all waiting and recovery periods indicating diminished vagal activity during stress. This result is in accordance to RMSSD in the time domain which also reflects parasympathetic activity. Furthermore, HF% was also low during stress period T5 but it was not different from the waiting periods T1 and T2. Hence, in this case HF is superior compared to HF% because the results of HF are more consistent. Still, it does not consistently show a decrease of vagal activity during stress periods T4 and T5. LF/HF, sometimes denoted as sympathovagal balance^36^, was also not able to consistently reflect the sympathetic activation and parasympathetic deactivation during both stress periods. Taken together, the frequency domain parameters of HRV were not able to consistently reflect the imposed stress on cardiovascular regulation. The time domain parameter SDNN was more consistent in this respect.

The results of the parameters in the time and frequency domain are in agreement with recent findings. Acute mental stress exerted by a mental arithmetic task could be reliably quantified using RMSSD, LFnu and HFnu (i.e. LF and HF expressed in normalized units as an equivalent to HF% and LF% in the present study) and HF^29^. LF was not able to reliably quantify the acute stress. The present results suggest that the effect of nonstationarities in the RR interval series may have contributed to this result.

The parameters derived from symbolic dynamics are not biased by nonstationary RR interval series because the associated series of differences between successive RR intervals is stationary although the underlying RR interval series may be nonstationary. P0V% and P0V_τ_% consistently increased during stress periods T4 and T5 compared to waiting and recovery periods. It has been shown that P0V% and P0V_τ_% may be interpreted in terms of sympathetic activity, i.e. the higher P0V% and P0V_τ_% the higher the sympathetic activity^21,22,37^. Hence, these parameters indicate the time course of low sympathetic activity during waiting and recovery periods as well as sympathetic activation during acute stress. On the other hand, P1V% and P1V_τ_% consistently decreased during stress and were lower compared to waiting and recovery periods. As these parameters indicate parasympathetic activity, i.e. the higher P1V% and P1V_τ_% the higher the parasympathetic activity, also the course of parasympathetic activity could be clearly captured by these parameters. P2V% and P2V_τ_% also decreased during stress periods T4 and T5 compared to the waiting and recovery periods. However, these pattern categories could not be unambiguously linked to sympathetic or parasympathetic activity^21,22,37^. Hence, these parameters remain unclear with respect to the interpretation in terms of cardiac autonomic regulation.

We note that gender differences were only observed during the stress period T4: although the perceived stress was similar, male subjects had a higher median RR interval compared to female subjects. However, this difference did not affect e.g. the amount of nonstationarities nor did it affect any parameter in the time and frequency domain or parameters from the symbolic dynamics analysis. Recent studies showed that female subjects had higher perceived stress and lower HRV parameters compared to male subjects^28^. These differences could be attributed to gender differences in e.g. coping styles and emotion regulation strategies^38^. In the present study gender differences were not observable most likely due to the relatively homogenous study population (University students) and the participation dates of female subjects that had to be in the second half of their menstrual cycle to decrease gender differences^39^.

In conclusion, nonstationarities in RR interval series occur during transient states like e.g. acute stress but our analysis showed that also during resting and quiet states nonstationarities have to be expected. From this perspective, the quantification of HRV with frequency domain parameters is limited in almost any case because the requirement of stationarity is rarely met. The nonstationarities obviously biased the LF and HF and, as a result, these parameters were less informative than e.g. the RR interval series or SDNN because the latter parameters clearly indicated acute stress whereas LF and HF did not. Methods based on the differences of RR intervals like e.g. parameters derived from binary symbolic dynamics are more informative compared to frequency domain parameters. They do not depend on nonstationarities and they can also be interpreted in terms of cardiac autonomic regulation and, hence, allow physiologically meaningful interpretations.

## Methods

This study is part of a multi-faceted research project, investigating different aspects of stress-responsive processes to gain a comprehensive approach to acute psychological stress perception and response. We investigated psychological parameters of subjective stress perception, physiological stress parameters such as HRV-parameters, biochemical stress parameters (salivary cortisol levels and salivary alpha-amylase activity) as well as epigenetic parameters such as salivary microRNAs (miRNAs). For further details on other aspects of this project, we would like to refer to our recent publications^8,9^.

### Subjects

24 healthy subjects (12 female, age: 20–35 years) were recruited among a population of University students. The female subjects had to be in the second part of their menstrual cycle at the date of participation because sex differences were observed in stress responsibility which apply mainly to the first half of the menstrual cycle^39^. Furthermore, female participants did not take any hormonal contraception. All participants were in good physical and psychological health, had no history of psychiatric diseases, were non-smoking, taking no drugs, alcohol or medication, and did not perform any type of meditation or relaxation-exercises regularly (more than once a week). The female participants did not take any hormonal contraception and were in the second half of their menstrual cycle at the date of participation. To minimize interference with circadian variations of cortisol levels, all tests were carried out between 3 and 5 p.m^9^.

All experiments were conducted in accordance with the Declaration of Helsinki. The study was approved by the ethical committee of Witten/Herdecke University, Germany (96/2015) and registered in the German Register for Clinical Studies DRKS which is linked to the WHO-Register (Registration-ID: DRKS00010134). The participants were informed in a written and oral format about the study aims and procedures, particularly their participation in a psychosocial stress test, and the timetable of the experiment was explained. To prevent reduced stress reactions due to prior mental adaption to the expected task, participants were not informed about the specific details of the TSST. Written informed consent of each participant was obtained before the onset of the experiment. They were debriefed at the end of the experiment receiving full information about the procedure^9^.

### Experimental stress test

**Figure 2:**
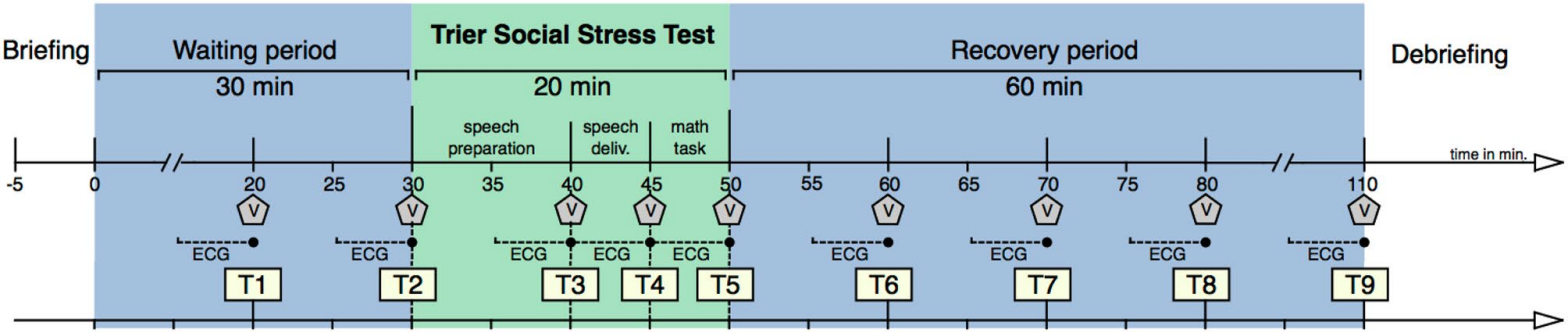
Experimental design and time-course. T1, …, T9 denote the analysis periods (duration: 5 min) and V denotes the times of assessment of perceived stress on the visual analogue scale.

The Trier Social Stress Test (TSST)^10^ was applied as a reliable and standardized acute psychosocial stress test, mainly following the TSST-protocol by Birkett, 2011. Time-points of data collection were modified to suite the individual requirements of the study (Fig. 2). The stress test was split in three periods: a waiting period of 30 min, the actual stress test period of 20 min, and a recovery period of 60 min. During the whole procedure, the participants were not allowed to use any electronic media. During the waiting and recovery period, the participants could relax being located on their own in a quiet atmosphere with comfortable seating.

The actual TSST test period took place in the social laboratory, a simply equipped room containing a chair for the participant during the preparation phase as well as an office desk and two chairs for the ‘experts’. There, the participants had to prepare (10 min) and then deliver (5 min) an oral presentation applying for an individually ideal job-offer. Afterwards, they had to perform a mental arithmetic task (5 min), sequentially subtracting 13 from 1,022. The speech delivery and mental arithmetic task took place in front of a panel of two persons as ‘experts’, who followed a strict protocol. They wore white lab coats, exhibited unemotional neutrality, avoided any oral or mimic feedback, and just adverted if there was still time remaining or if a mistake was made during the mental arithmetic task, instructing the participant to start again from 1,022. Furthermore, dummies of a video camera and a microphone were installed and the participants were told to be recorded during their speech delivery and mental arithmetic task.

### Subjectively perceived stress

A visual analogue scale (VAS) ranging from 0 (feeling no stress at all) to 100 (feeling maximally stressed) was used to assess the subjectively perceived stress at nine time points; before (T1 + T2), during (T3–T5) and after (T6–T9) the actual stress test^40^ (Fig. 1).

### Heart rate variability

An electrocardiogram (ECG) was recorded continuously throughout the procedure using a portable Holter recorder (TOM Medical MK3, Graz, Austria). Time markers were set concurrently to the VAS at nine time points (T1, … T9) to enable proper identification of the previous 5 min as respective analysis periods (Fig. 1).

The Holter device’s sampling rate of the ECG was 4,096 Hz. Hence, the internally detected times of R-peaks had a precision < 1 ms. The times of the R-peaks and the ECG at a sampling rate of 256 Hz were saved on a memory card. The detected times of R-peaks were visually checked and corrected in case of false detections due to e.g. artifacts (< 1% of all detected R-peaks). Subsequently, the RR interval series was calculated as the temporal distance between successive R-peaks. Times of ventricular and supraventricular beats were replaced by appropriately interpolated times^41^. The resulting RR interval series $RR_{i} \left( {i = 1, \ldots ,N} \right)$ served as the basis for the HRV analysis.

The RR interval series of each 5 min analysis period was quantified as follows. The average RR interval, its standard deviation (SDNN) and the root of the squared mean difference between successive RR intervals (RMSSD) were calculated as basic parameters in the time domain. The median length of the RR interval series varied between 338 (T9) and 483 (T4) RR intervals. The calculation of the frequency domain parameters was carried out using a re-sampled time series at 4 Hz. The re-sampled time series was detrended and a Hanning window was applied. Low (LF: 0.04–0.15 Hz) and high frequency (HF: 0.15–0.4 Hz) oscillations and the fraction LF/HF were quantified using a fast Fourier transformation (2048 data points with zero padding)^17^. The total spectral power was adjusted to the variance of the RR interval series and, hence, HF and LF were expressed in ms^2^. The proportions of LF and HF in relation to the total power, LF% and HF%, were also calculated because these quantities tend to minimize the impact of changes in total power^17^. Hence, they may better reflect changes of cardiac autonomic regulation^21,35^.

### Symbolic analysis

The RR interval series $RR_{i} \left( {i = 1, \ldots ,N} \right)$ is transformed into a binary symbolic series by two different approaches. The first approach simply reflects the succession of acceleration and deceleration of heart rate. I.e. the difference series $\Delta RR_{i} = RR_{i} - RR_{i - 1} \left( {i = 2, \ldots ,N} \right)$ is calculated and the symbolic sequence is created according to the sign of each difference^37^:

$$ S_{i} = \left\{ {\begin{array}{*{20}l} {0,} \hfill & {\quad {\text{if}}{\mkern 1mu} {\mkern 1mu} \Delta RR_{i} \ge 0} \hfill \\ {1,} \hfill & {\quad {\text{if}}{\mkern 1mu} {\mkern 1mu} \Delta RR_{i} < 0} \hfill \\ \end{array} } \right. $$

**Figure 3:**
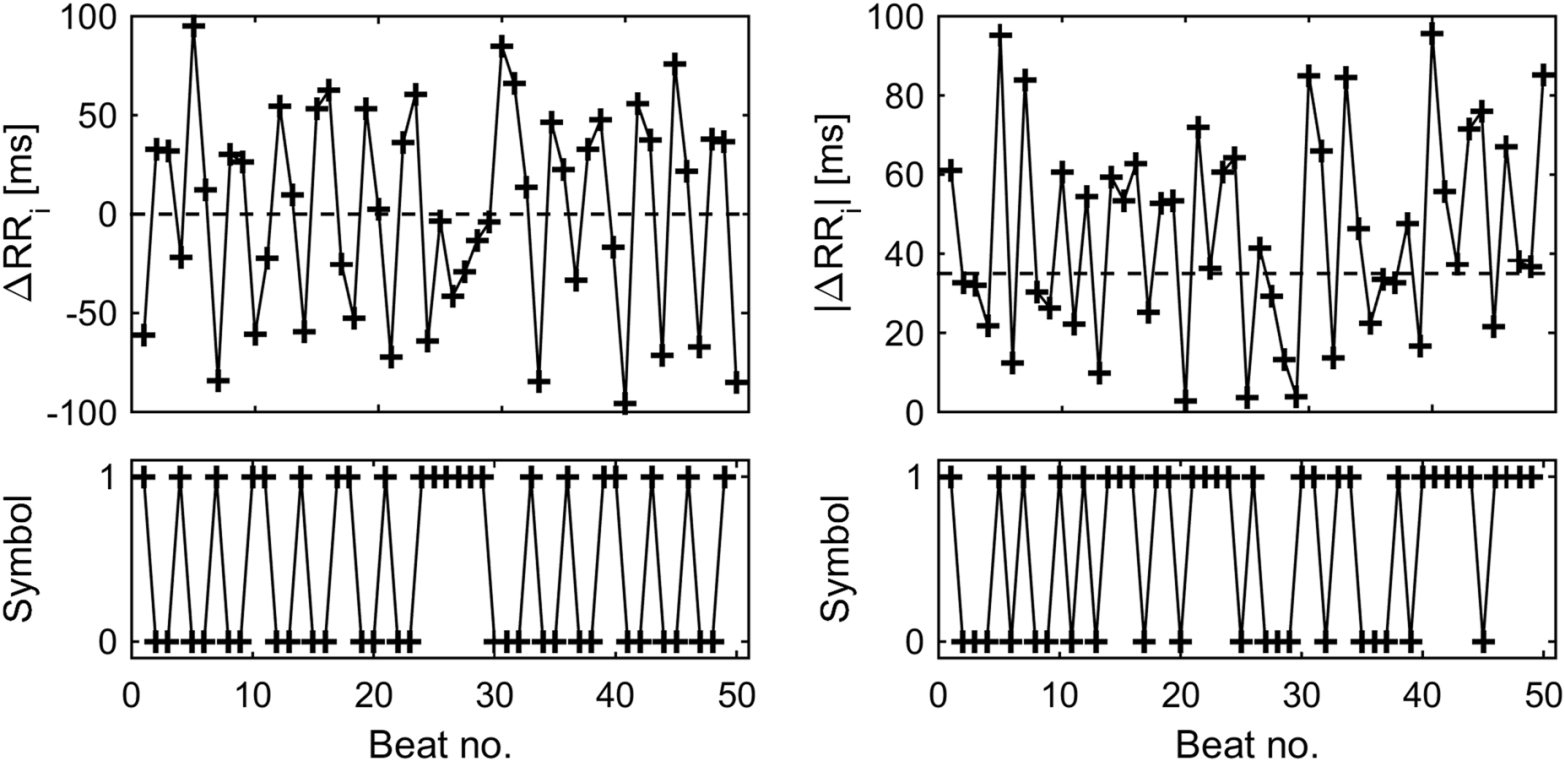
Construction of symbolic sequences. Examples of the construction of binary sequences *S*_*i*_ (left column) and *S*_*τ,i*_ (right column) taken from one subject during analysis period T7. The dashed lines indicate the thresholds for the assignment of 0 s and 1 s. The sequences *S*_*i*_ und *S*_*τ,i*_ contain considerably different successions of 0 s and 1 s.

The 0 s represent decelerations of the heart rate whereas the 1 s represent accelerations (see Fig. 3, left column). In this approach, no parameter has to be chosen.

In the second approach the difference series Δ*RR*_*i*_ is transformed into a binary series according to a pre-defined threshold. The binary coding represents whether the absolute value of the difference Δ*RR*_*i*_ is below or above the threshold *τ*:

$$ S_{\tau ,i} = \left\{ {\begin{array}{*{20}l} {0,} \hfill & {\quad {\text{if}}{\mkern 1mu} {\mkern 1mu} \left| {\Delta RR_{i} } \right| < \tau } \hfill \\ {1,} \hfill & {\quad {\text{if}}{\mkern 1mu} {\mkern 1mu} \left| {\Delta RR_{i} } \right| \ge \tau } \hfill \\ \end{array} } \right. $$

This transformation reflects whether the succession of RR intervals has only small changes (0 s) or also contains larger changes (1 s, see Fig. 3, right column). In this study, the threshold *τ* is set to 35 ms, i.e. approximately 5% of the grand average RR interval. This threshold resulted in binary series with sufficient changes between 0 and 1 s reflecting changes in the dynamics of the RR interval series.

The symbolic sequences *S*_*i*_ and *S*_*τ,i*_ were analyzed with respect to the amount of variations between successive symbols in the binary sequence. All subsequences of length *k* = 3, i.e. 2^3^ = 8 binary sequences, are categorized as follows:

- 0 V sequences: no variations between three successive symbols, i.e. all three symbols are equal (‘000’ and ‘111’).
- 1 V sequences: one variation between three successive symbols, i.e. two symbols are equal (‘001’, ‘100’, ‘110’ and ‘011’).
- 2 V sequences: two variations between successive symbols (‘101’ and ‘010’).

We calculated the relative frequency of each pattern category for both symbolic sequences (P0V%, P1V%, P2V% and P0V_τ_%, P1V_τ_%, P2V_τ_%). It has been shown that the categories P0V%, P0V_τ_% and P1V%, P1V_τ_% properly reflect sympathetic and parasympathetic modulations of cardiac autonomic regulation, respectively^22,37^.

### Analysis of nonstationarities in the RR interval series

Methods such as the Fourier transformation to calculate LF and HF require stationarity of the analyzed time series. A time series is stationary if its statistical characteristics (e.g. the mean, the standard deviation and all higher moments) are invariant with respect to time translation. However, especially in time series like the RR interval series during stress this prerequisite is seldom met. To quantify the amount of nonstationarities in each analysis period T1, …, T9 two different approaches are used. The first approach uses a heuristic segmentation of the time series. Stationary segments are created utilizing the pooled variance of two adjacent segments in such a way that the mean value between adjacent segments is maximized^42,43^. We set the minimum length of stationary segments to 40 RR intervals because shorter segments could lead to a segmentation caused by fluctuations in the low frequency band. The amount of segments in each analysis period is used as an indicator of nonstationarities. In a stationary analysis period the RR interval series must not be segmented.

The second approach is called the restricted weak stationarity (RWS) test and checks the mean and the variance of randomly chosen subsequences of the time series under consideration^44^. In case of stationarity all subsequences should have the same mean and variance. This approach quantifies separately the probability of different means and different variances in the subsequences. In stationary RR interval series these probabilities should be *p* > 0.05. As suggested by the authors we used 8 randomly subsequences containing 50 RR intervals for this approach.

### Statistical analysis

All statistical procedures are descriptive. Non-parametric statistical procedures were used because of the low number of subjects. The distributions of LF, HF and LF/HF showed skewed distributions and, hence, they were transformed taking the natural logarithm. The distribution of each parameter was quantified by the median and the interquartile range (25% and 75%-percentile). Non-parametric statistical procedures were consistently used. The Friedman test for repeated measures was used to assess changes of the parameters at the times T1–T9. In case of missing values (VAS), the Skillings-Mack test was used as a replacement for the Friedman test to take advantage of all available data^45^. If the Friedman test (or the Skillings-Mack test) showed significant changes of a parameter, pair-wise differences between different times were checked post-hoc including adjustment for multiple comparisons^46^. Gender differences were tested using the Mann–Whitney U-test. A *p* < 0.05 was considered statistically significant.

## References

[1] Cohen S, Janicki-Deverts D, Miller GE (2007). Psychological stress and disease. JAMA.

[2] Cohen S, Kessler RC, Gordon LU, Cohen S, Kessler RC, Gordon LU (1995). Conceptualizing stress and its relation to disease. Measuring Stress: A Guide for Health and Social Scientists.

[3] Cohen S, Tyrrell DA, Smith AP (1991). Psychological stress and susceptibility to the common cold. N. Engl. J. Med..

[4] Gu HF, Tang CK, Yang YZ (2012). Psychological stress, immune response, and atherosclerosis. Atherosclerosis.

[5] Stenstrom U, Wikby A, Hornquist JO, Andersson PO (1993). Recent life events, gender, and the control of diabetes mellitus. Gen. Hosp. Psychiatry.

[6] Yonas MA, Lange NE, Celedon JC (2012). Psychosocial stress and asthma morbidity. Curr. Opin. Allergy Clin. Immunol..

[7] Salleh MR (2008). Life event, stress and illness. Malays. J. Med. Sci..

[8] Wiegand C, Savelsbergh A, Heusser P (2017). MicroRNAs in psychological stress reactions and their use as stress-associated biomarkers, especially in human saliva. Biomed. Hub.

[9] Wiegand C (2018). Stress-associated changes in salivary microRNAs can be detected in response to the Trier Social Stress Test. An exploratory study. Sci. Rep..

[10] Kirschbaum C, Pirke KM, Hellhammer DH (1993). The 'Trier Social Stress Test'—A tool for investigating psychobiological stress responses in a laboratory setting. Neuropsychobiology.

[11] Birkett MA (2011). The Trier Social Stress Test protocol for inducing psychological stress. J. Vis. Exp..

[12] Allen AP, Kennedy PJ, Cryan JF, Dinan TG, Clarke G (2014). Biological and psychological markers of stress in humans: Focus on the Trier Social Stress Test. Neurosci. Biobehav. Rev..

[13] Lucassen PJ (2014). Neuropathology of stress. Acta Neuropathol..

[14] Frisch JU, Hausser JA, Mojzisch A (2015). The Trier Social Stress Test as a paradigm to study how people respond to threat in social interactions. Front. Psychol..

[15] Pereira T, Almeida PR, Cunha JPS, Aguiar A (2017). Heart rate variability metrics for fine-grained stress level assessment. Comput. Methods Programs Biomed..

[16] Nater UM (2006). Stress-induced changes in human salivary alpha-amylase activity—Associations with adrenergic activity. Psychoneuroendocrinology.

[17] Task Force of the European Society of, C., the North American Society of, P. & Electrophysiology. Heart rate variability: standards of measurement, physiological interpretation, and clinical use. *Circulation* **93**, 1043–1065 (1996). 8598068

[18] Castaldo R, Montesinos L, Melillo P, James C, Pecchia L (2019). Ultra-short term HRV features as surrogates of short term HRV: A case study on mental stress detection in real life. BMC Med. Inform. Decis. Mak..

[19] McNames J, Aboy M (2006). Reliability and accuracy of heart rate variability metrics versus ECG segment duration. Med. Biol. Eng. Comput..

[20] Singh D, Vinod K, Saxena SC, Deepak KK (2004). Effects of RR segment duration on HRV spectrum estimation. Physiol. Meas..

[21] Cysarz D, Van Leeuwen P, Edelhäuser F, Montano N, Porta A (2012). Binary symbolic dynamics classifies heart rate variability patterns linked to autonomic modulations. Comput. Biol. Med..

[22] Cysarz D (2013). Quantifying heart rate dynamics using different approaches of symbolic dynamics. Eur. Phys. J. Spec. Top..

[23] Bernardi L (2000). Effects of controlled breathing, mental activity and mental stress with or without verbalization on heart rate variability. J. Am. Coll. Cardiol..

[24] Houtveen JH, Rietveld S, de Geus EJ (2002). Contribution of tonic vagal modulation of heart rate, central respiratory drive, respiratory depth, and respiratory frequency to respiratory sinus arrhythmia during mental stress and physical exercise. Psychophysiology.

[25] Vuksanovic V, Gal V (2007). Heart rate variability in mental stress aloud. Med. Eng. Phys..

[26] Kuehl LK (2015). Two separable mechanisms are responsible for mental stress effects on high frequency heart rate variability: An intra-individual approach in a healthy and a diabetic sample. Int. J. Psychophysiol..

[27] Klinkenberg AV (2009). Heart rate variability changes in pregnant and non-pregnant women during standardized psychosocial stress. Acta Obstet. Gynecol. Scand..

[28] Brugnera A (2018). Heart rate variability during acute psychosocial stress: A randomized cross-over trial of verbal and non-verbal laboratory stressors. Int. J. Psychophysiol..

[29] Chen Y, Zhang L, Zhang B, Chan CA (2020). Short-term HRV in young adults for momentary assessment of acute mental stress. Biomed. Signal Process. Control.

[30] Schiweck C, Piette D, Berckmans D, Claes S, Vrieze E (2019). Heart rate and high frequency heart rate variability during stress as biomarker for clinical depression. A systematic review. Psychol. Med..

[31] Wilhelm FH, Grossman P, Roth WT (2005). Assessment of heart rate variability during alterations in stress: Complex demodulation vs. spectral analysis. Biomed. Sci. Instrum..

[32] Cysarz D, Büssing A (2005). Cardiorespiratory synchronization during Zen meditation. Eur. J. Appl. Physiol..

[33] Eckberg DL, Kuusela TA (2005). Human vagal baroreflex sensitivity fluctuates widely and rhythmically at very low frequencies. J. Physiol..

[34] Houtveen JH, Molenaar PC (2001). Comparison between the Fourier and Wavelet methods of spectral analysis applied to stationary and nonstationary heart period data. Psychophysiology.

[35] Montano N (1994). Power spectrum analysis of heart rate variability to assess the changes in sympathovagal balance during graded orthostatic tilt. Circulation.

[36] Eckberg DL (1997). Sympathovagal balance: A critical appraisal. Circulation.

[37] Cysarz D (2015). Symbolic transformations of heart rate variability preserve information about cardiac autonomic control. Physiol. Meas..

[38] Kelly MM, Tyrka AR, Anderson GM, Price LH, Carpenter LL (2008). Sex differences in emotional and physiological responses to the Trier Social Stress Test. J. Behav. Ther. Exp. Psychiatry.

[39] Kirschbaum C, Kudielka BM, Gaab J, Schommer NC, Hellhammer DH (1999). Impact of gender, menstrual cycle phase, and oral contraceptives on the activity of the hypothalamus-pituitary-adrenal axis. Psychosom. Med..

[40] Hellhammer DH, Wust S, Kudielka BM (2009). Salivary cortisol as a biomarker in stress research. Psychoneuroendocrinology.

[41] Wessel N (2000). Nonlinear analysis of complex phenomena in cardiological data. Herzschrittmacherther. Elektrophysiol..

[42] Bernaola-Galvan P, Ivanov PC, Nunes Amaral LA, Stanley HE (2001). Scale invariance in the nonstationarity of human heart rate. Phys. Rev. Lett..

[43] Fukuda K, Stanley HE, Amaral LAN (2004). Heuristic segmentation of a nonstationary time series. Phys. Rev. E.

[44] Porta A, D'Addio G, Guzzetti S, Lucini D, Pagani M (2004). Testing for the presence of nonstationarities in short heart rate variability series. Comput. Cardiol..

[45] Skillings JH, Mack GA (1981). On the use of a Friedman-type statistic in balanced and unbalanced block designs. Technometrics.

[46] Conover WH (1999). Practical nonparametric statistics.

